# Group Facial Width-to-Height Ratio Predicts Intergroup Negotiation Outcomes

**DOI:** 10.3389/fpsyg.2018.00214

**Published:** 2018-02-21

**Authors:** Yu Yang, Chen Tang, Xiaofei Qu, Chao Wang, Thomas F. Denson

**Affiliations:** ^1^School of Entrepreneurship and Management, ShanghaiTech University, Shanghai, China; ^2^School of Labor and Employment Relations, University of Illinois at Urbana-Champaign, Champaign, IL, United States; ^3^Department of Business Administration, College of Business, University of Illinois at Urbana-Champaign, Champaign, IL, United States; ^4^School of Psychology, University of New South Wales, Sydney, NSW, Australia

**Keywords:** facial width-to-height ratio, FWHR, intergroup negotiation, team dynamics, group behavior

## Abstract

Past studies have found that the facial width-to-height ratio (FWHR) is associated with a range of traits and behaviors that are possibly important to dyadic negotiations. However, it is unknown whether the FWHR would have an impact on intergroup negotiations, which happen frequently and often have higher stakes in the real world. To examine this question, in the current study, we randomly assigned 1,337 Chinese business executives into 288 groups and they completed a multi-issue negotiation exercise against each other. Results showed that groups with larger maximum individual FWHRs achieved objectively better negotiation outcomes. We conclude that groups containing individuals with relatively large FWHRs can claim more value in negotiations between groups.

## Introduction

People often have to make quick decisions about strangers based on very little information (Ambady et al., 2000). When meeting unfamiliar people, being able to quickly recognize who may help or hurt would have conferred survival and reproductive advantages to our ancestors. In today’s cosmopolitan world of cities with millions of strangers speaking hundreds of languages, this ability is arguably even more useful. People often negotiate with strangers who are motivated to maximize personal and group outcomes. In the negotiation context, negotiators would be at an advantage if they could quickly and accurately gauge aspects of their negotiation partners’ characteristics.

The face is used for social communication and is therefore a particularly useful tool for inferring individual characteristics. The ratio of the face’s width to height, which is called the facial width-to-height ratio (FWHR), is one cue that perceivers implicitly use to determine the extent to which men might be trustworthy, honest, dominant, or aggressive (Carré et al., 2009; McCormick et al., 2010; Stirrat and Perrett, 2010; Geniole et al., 2012). We focused on men in our current analysis because a meta-analysis reported that whereas men with relatively large FWHRs are perceived as dominant, this effect is not as robust in women (Weston et al., 2007; Lefevre et al., 2013; Geniole et al., 2015). Two recent meta-analyses also found that overall, men with larger FWHRs tend to behave in a dominant and aggressive manner and are perceived accordingly (Geniole et al., 2015; Haselhuhn et al., 2015). Men with larger FWHRs are perceived as more threatening, less human, and more animal-like (Geniole et al., 2015; Deska and Hugenberg, 2018; Deska et al., 2018). Thus, groups who negotiate with groups with relatively large FWHRs are likely to detect these intimidating qualities and concede more easily in negotiations against counterparts with relatively large FHWRs.

These traits associated with the FWHR can be important in negotiations. Emerging research shows that men with larger FWHRs are more likely to deceive others during a dyadic negotiation, cheat, and exploit the trust of others (Stirrat and Perrett, 2010; Haselhuhn and Wong, 2012; Geniole et al., 2014). Similarly, when participants ostensibly interacted with men with high FWHRs, they reacted by protecting their resources during dyadic resource allocation games (Haselhuhn et al., 2013). Most recently, in the context of interindividual, dyadic negotiations, men with larger FWHRs claimed more value and created less value than men with smaller FWHRs (Haselhuhn et al., 2014). Perhaps due to the intimidating perceptions associated with a large FWHR, groups prefer leaders with a larger FWHR (Hehman et al., 2015). Thus, it is possible that just one group member with a large FWHR can increase the value claimed during a negotiation.

Furthermore, there is some suggestive evidence that group leaders with larger FWHRs may enjoy greater success in business and politics. For instance, one study of 55 white male CEOs from Fortune 500 companies found that larger CEO FWHRs positively correlated with the company’s financial success (Wong et al., 2011). Another study of former US presidents (all White) found that presidents with larger FWHRs were rated as higher in achievement motivation and lower in social graces (Lewis et al., 2012). Thus, at least among group leaders, a larger FWHR may partially determine the group’s success. However, these correlational findings leave open the possibility that more successful groups or events during certain historical periods cause group members to prefer leaders with larger FWHRs. Research with random assignment to groups is needed.

To date, no study has examined whether the FWHR influences negotiations between groups. Negotiation researchers have long argued that intergroup negotiations, such as those between companies and organizations, happen frequently and have higher stakes in the real world than dyadic negotiations (Thompson et al., 1996; Brodt and Tuchinsky, 2000). They have also shown that negotiators often behave differently in dyadic versus in intergroup situations (e.g., Thompson et al., 1996; O’Connor, 1997; Morgan and Tindale, 2002; Wildschut et al., 2003; Gelfand et al., 2013). Hence, it remains an empirical question whether the effects of the FWHR established at the dyadic level may or may not replicate at the intergroup level.

Because people with large FWHRs are perceived as threatening, dominant, and animal-like, we expected that groups with relatively larger FWHRs (either a single individual or the group’s average FWHR) would claim relatively greater value in the negotiation (Geniole et al., 2015; Deska and Hugenberg, 2018; Deska et al., 2018). These qualities should be intimidating to their negotiation counterparts. However, it is also plausible that group processes may increase or decrease the ability of men with relatively large FWHRs to claim value within negotiations. On the one hand, compared to dyadic negotiations, men with relatively larger FWHRs may find their influence attenuated in intergroup situations. For instance, other group members with relatively smaller FWHRs may less effectively compete for resources and thereby weaken the overall negotiation effectiveness of their groups. It is also possible that men with larger FWHRs could have a detrimental effect on team dynamics and cohesion due to conflict over their dominant and aggressive behavior. On the other hand, groups with larger FWHRs on average may negotiate better deals because of the added dominance in their groups when they compete with other groups with smaller average FWHRs. If the groups consist of members with similarly large FWHRs, they might be able to better coordinate their actions in negotiations because of the similarity among them in terms of dominant personality. Furthermore, within groups, men with larger FWHRs may be better able to obtain leadership positions within their groups. Their influence, therefore, may be amplified. Thus, this research investigated the extent to which the FWHR may have a positive or negative impact on intergroup negotiation outcomes.

Moreover, research on intergroup negotiation has typically focused on situational characteristics associated with the negotiation task (e.g., Mannix et al., 1989; Mannix, 1993), team members (e.g., Peterson and Thompson, 1997; Beersma and De Dreu, 1999; Gelfand and Realo, 1999; Schei and Rognes, 2005), or both (e.g., Weingart et al., 1993; Ten Velden et al., 2007). With the exception of negotiator sex (Kray and Thompson, 2004; Gladstone and O’Connor, 2014), few studies have aimed to identify relatively stable characteristics of negotiators that may influence negotiation outcomes. The present study therefore makes a novel contribution by examining whether the largely static characteristic of the FWHR influences negotiations at the intergroup level.

Finally, the FWHR may be a universal cue through which people make social inferences about dominance, aggression, and trustworthiness (Christiansen and Winkler, 1992; Short et al., 2012). Even 8-year-old Caucasian and Chinese children make use of the FWHR to infer aggressiveness (Short et al., 2012). Empirical examination of the potential effect of the FWHR outside of the West can be particularly valuable as the FWHR is thought to be a universal cue of dominance. If use of the FWHR is ubiquitous, then we should see universal evidence of its utility. To our knowledge, only one investigation has examined the role of the FWHR in non-White samples (Short et al., 2012) and no study has examined the FWHR within the context of intergroup negotiations. To address these gaps in our knowledge, in the present study, we randomly assigned Chinese business executives into groups and they completed a multi-issue negotiation exercise against each other to earn points. We found that groups with larger maximum individual FWHRs earned more points in the negotiation.

## Materials and Method

### Participants and Design

A total of 1,337 Chinese Executive MBA students (*M*_age_ = 40.67, *SD*_age_ = 4.74) were randomly assigned to 288 teams of four or five people and participated in an intergroup negotiation between buyers and sellers as part of a class exercise. Sample size was determined by the class size. Each team was also assigned a team role of buyers or sellers. The data were collected across two semesters in a major Chinese business school at its Beijing (Northern China), Shanghai (Eastern China), and Shenzhen (Southern China) campuses. The second wave of participants (*n* = 697) used a negotiation exercise slightly revised from the one used by the first wave of participants (*n* = 640). The payoff schedules in the two exercises were the same (see **Table 1**) and we controlled for exercise throughout our data analysis. The independent variables were the team’s average FWHR and maximum individual FWHR, as the two variables are central for testing the role that the FWHR might play in intergroup negotiations. The dependent variables were the number of points earned in the negotiation (*M* = 6,572.12, *SD* = 1,060.40, converted to *z*-scores independently for each wave in our analyses).

**Table 1:** Payoff schedule in negotiation.

Issues and Options	Buyer	Seller
	payoff	payoff
**Issue 1: Technology**		
Option 1: Seller to transfer all	3,200	0
Option 2: Seller to transfer all but with limited usage	2,400	300
Option 3: Seller to transfer half	1,600	600
Option 4: Seller to transfer small portion	800	900
Option 5: Seller not to transfer	0	1,200
**Issue 2: Price**		
Option 1: 458k/day	0	3,200
Option 2: 440k/day	300	2,400
Option 3: 422k/day	600	1,600
Option 4: 404k/day	900	800
Option 5: 386k/day	1,200	0
**Issue 3: Responsibility**		
Option 1: Seller 50% vs. Buyer 50%	4,000	0
Option 2: Seller 35% vs. Buyer 65%	3,000	1,000
Option 3: Seller 25% vs. Buyer 75%	2,000	2,000
Option 4: Seller 15% vs. Buyer 85%	1,000	3,000
Option 5: Seller 0% vs. Buyer 100%	0	4,000
**Issue 4: Emission**		
Option 1: 0.5ug/m^3^	1,600	1,600
Option 2: 0.4ug/m^3^	1,200	1,200
Option 3: 0.3ug/m^3^	800	800
Option 4: 0.2ug/m^3^	400	400
Option 5: 0.1ug/m^3^	0	0

### Materials and Procedure

Teams were given 35 min to prepare for the negotiation in their own teams and 45 min to complete the negotiation with their counterparts. The negotiation exercise involved negotiating the terms of an engineering project contract between a buyer and a seller. During the preparation time, teams were provided with a description of the negotiation and a payoff matrix outlining the points awarded for each issue (see **Table 1**). Teams engaged in a mixed-motive negotiation with one distributive issue, two integrative issues, and one compatible issue, where both parties had identical interests. Distributive issues are zero sum as both parties have opposing interests. Integrative issues provide the opportunity to create value by trading off on issues. The study was conducted in compliance with APA ethical standards and was approved by the Human Research Ethics Committee of ShanghaiTech University as part of a larger research project examining background information of the students and negotiation outcomes produced in class exercises. Because the study was part of a class exercise, the ethics committee provided exemption from obtaining informed consent.

### FWHR Measurement and Data Exclusion

**FIGURE 1:**
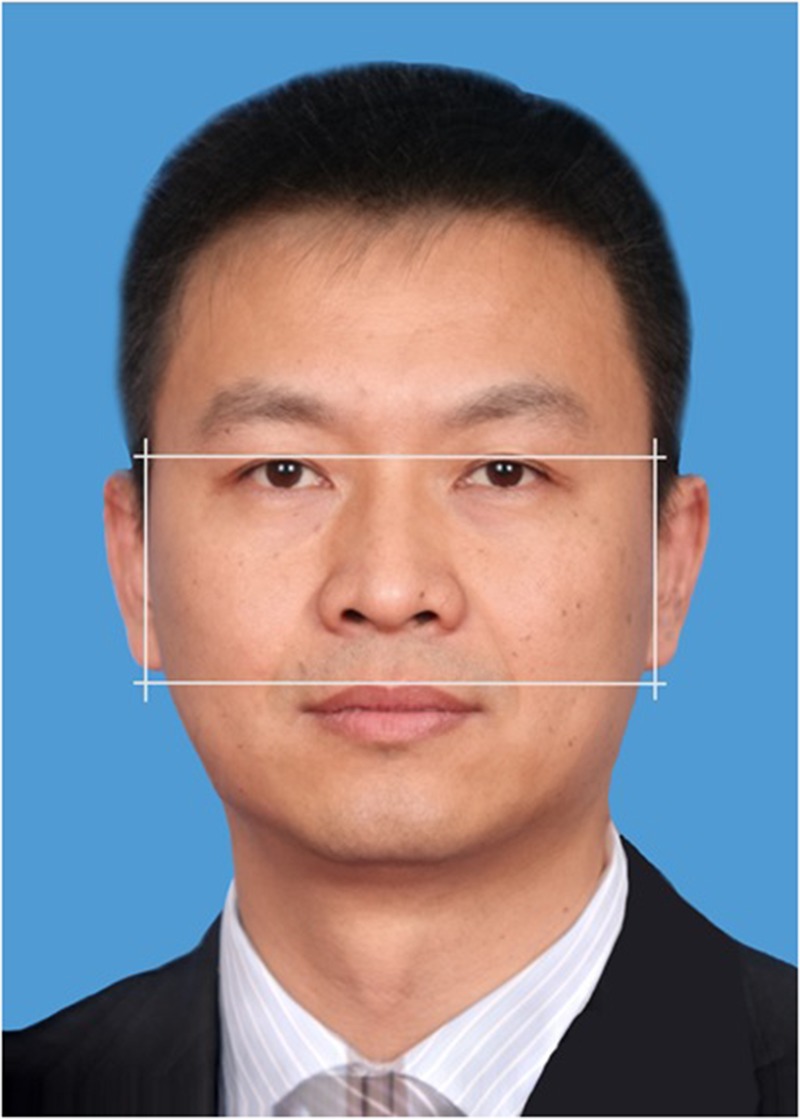
Example of measuring FWHR. Vertical lines represent the distance between the left and the right zygion (bizygomatic width). Horizontal lines represent the distance between the highest point of the upper lip and the highest point of the eyelids (facial height). This photo is for illustrative purpose only and does not depict an actual participant in the study. To protect the identity of participants, we produced the present photo by merging three photos using Psychomorph (Tiddeman et al., 2005).

As part of the process of enrolling in the Executive MBA program, participants were photographed front-on and posed with a neutral expression. To calculate FWHR, the bizygomatic width of the face is divided by height of the face (see **Figure 1**). The bizygomatic width refers to the distance between the left and right zygon. The height of the face is measured from the highest point of the upper lip to the highest point of the eyelids (Carré and McCormick, 2008). An image-processing program was used to rotate the photographs so that the pupils were on the same transverse plane and the height and width was measured (Kramer et al., 2012). To calculate FWHR all facial boundaries need to be clearly visible. Following previous research, any participants with a tilted head, hair covering their face, visible teeth or asymmetry in their eyes or mouth were excluded from the data analyses (*n* = 150 males). We also excluded 44 very overweight individuals as their zygons could not be clearly identified. As a large number of the sample wore glasses (44%), data from participants with glasses were retained in the analyses (Carré et al., 2010). We did not deem glasses as problematic because the facial boundaries remained visible. Of all of the participants, ten did not provide their photographs. Female negotiators (*n* = 326) were excluded from the analyses because FWHR is a more reliable marker amongst men than women (e.g., (Carré and McCormick, 2008); Geniole et al., 2015). Following these exclusions, any teams with less than two participants were excluded from the analyses along with the opponent team (*n* = 101). Of the remaining 236 teams, ten were excluded because they reached impasses in the negotiation (*n* = 26). This left a total of 680 male negotiators in 226 teams. Two research assistants independently measured all the remaining photographs. Inter-rater agreements were high (α = 0.99 for facial width, α = 0.99 for height, and α = 0.99 for overall FWHR). We therefore averaged measurement of the two assistants for all FWHRs. Mean FWHR of the teams was 2.18 (*SD* = 0.08) and ranged from 1.99 to 2.50. Finally, we examined the photographs of all 326 women’s faces but only 123 (38%) could be measured for the FWHR because of head tilt, hair covering their face, or visible teeth or asymmetry of the eyes or mouth. Thus, we could not appropriately conduct analyses with women. However, including gender composition as a covariate showed that the number of women did not have systematic impact on our dependent variables.

## Results

### Data Analysis

Data were analyzed in R version 3.1.1 (R Core Team, 2016) with the packages ‘dplyr,’ ‘lavaan,’ ‘lme4,’ and ‘nlme’ (Rosseel, 2012; Bates et al., 2015; Pinheiro et al., 2016; R Core Team, 2016; Wickham et al., 2017). To control for the dyadic dependency in the data due to assignment to groups, we conducted linear mixed effects modeling using restricted maximum likelihood estimation. All analyses included a random intercept, which models variability in participant responses to the study nested within their teams. Including a random effects intercept assumes that not all participants were expected to respond in the same manner to the negotiation exercise. We also examined models with all random slopes, but these models did not fit the data better than the intercept-only models, *p*s > 0.99. We therefore report the results from the more parsimonious intercept-only model. In addition to maximum and average FWHR, our fixed effects covariates included the role that the teams were assigned and the wave of data collection. We ran separate models for maximum and average FWHR because these two variables were highly correlated, *r*(224) = 0.81, *p* < 0.0001. We also conducted sensitivity analyses. Specifically we included the proportion of women in the team and the average age of the team as covariates to observe if the effects of the FWHR were robust to age and gender composition.

We first examined the relationship between teams’ average FWHR and the total points that the teams negotiated (while controlling for role and wave of data collection). As shown in **Tables 2, 3**, teams’ average FWHR marginally predicted total points negotiated (*b* = 1.37, *SE* = 0.78, *t*[111] = 1.75, *p* = 0.083). Teams’ maximum individual FWHR significantly predicted total points (*b* = 1.14, *SE* = 0.57, *t*[111] = 2.02, *p* = 0.046). In both models, the role that each team played remained a significant predictor of points negotiated. Specifically, buyers earned more points than sellers, *t*s > 4.02, *p*s < 0.001. There were no other significant effects or interactions. The sensitivity analyses with average age and gender composition as covariates revealed the same pattern of results. The teams’ average FWHR remained a marginally significant predictor of total points negotiated (*b* = 1.31, *SE* = 0.78, *t*[109] = 1.67, *p* = 0.097) and the teams’ maximum FWHR remained a significant predictor of total points (*b* = 1.23, *SE* = 0.57, *t*[109] = 2.16, *p* = 0.033). In neither analysis did age or gender composition influence the negotiation outcomes, *p*s > 0.20.

**Table 2:** Average FWHR as predictor.

Variable	*b*	*SE*	*t* value	*p* value
Intercept	-2.14	2.10	-1.02	0.310
**Average FWHR**	**1.37**	**0.78**	**1.75**	**0.083**
Role	-0.52	0.13	-4.03	0.000
Exercise	0.00	0.07	-0.05	0.963

**Table 3:** Maximum FWHR as predictor.

Variable	*b*	*SE*	*t* value	*p* value
Intercept	-1.67	1.71	-0.98	0.330
**Maximum FWHR**	**1.14**	**0.57**	**2.02**	**0.046**
Role	-0.52	0.13	-4.02	0.000
Exercise	-0.01	0.07	-0.15	0.884

Separate analyses for the integrative issues revealed no significant effect of mean FWHR (*b* = 0.16, *SE* = 0.74, *t*[111] = 0.21, *p* = 0.831) or maximum FWHR (*b* = -0.13, *SE* = 0.54, *t*[111] = -0.24, *p* = 0.814). For distributive issues, there was no effect of mean FWHR on points (*b* = 1.02, *SE* = 0.77, *t*[111] = 1.31, *p* = 0.192), but there was a significant effect of maximum FWHR on distributive outcomes (*b* = 1.16, *SE* = 0.56, *t*[111] = 2.08, *p* = 0.040). These effects remained the same when controlling for age and gender composition.

An alternative way to analyze intergroup data is with the Actor-Partner Interdependence Model (APIM; Kashy and Kenny, 2000). Similar to our earlier analysis, APIM also takes into account the interdependence between two groups. Indeed, APIM can be implemented with linear mixed modeling (e.g., Campbell and Kashy, 2002; Wickham and Knee, 2012). The main feature of APIM is that it can distinguish the actor effect (e.g., the effect of FWHR on their own outcomes) and the partner effect (e.g., the effect of FWHR on their counterparts’ outcomes), as well as the interaction of the two effects. We reran the analysis using APIM as a supplementary analysis. The procedure suggested by Kenny and Ledermann (2010) and Fitzpatrick et al. (2016) is as follows: (1) fit the basic saturated model; (2) test the distinguishability of dyads to determine whether to employ the model for distinguishable or indistinguishable dyads; (3) estimate the effects using the suitable model; and (4) identify the pattern of the model, for example, identify whether it is an actor effect only model (partner effect does not exist), or whether actor effect equals the partner effect, or whether the actor effect and partner effect have the same value but with opposite signs.

In the APIM, we included two dependent variables: *z*-scores of the total points the buyers and the sellers negotiated. We also included two independent variables: the FWHR of buyers and sellers (teams’ average FWHR or maximum individual FWHR depending on the model) and the interaction between these independent variables.

We first examined the relationship between teams’ average FWHR and the total points that the teams negotiated. The test of distinguishability showed that the model for indistinguishable dyads fitted our data better (χ2(3) = 2.00, *p* = 0.573). Similar to the results using the linear mixed modeling, the teams’ average FWHR significantly positively predicted the points they negotiated (actor effect), *b* = 1.42, bootstrap 95% CI = [0.14, 2.98], but teams’ average FWHR did not predict the points their counterparts got (partner effect), *b* = -1.07, bootstrap 95% CI = [-2.66, 0.32]. The actor-partner interaction was not significant, *b* = 1.67, bootstrap 95% CI = [-7.86, 9.15]. Thus APIM largely replicated the results we obtained from the linear mixed model.

The results of teams’ maximum individual FWHR were slightly different. We also chose the model for indistinguishable dyads (χ2(3) = 1.89, *p* = 0.595). Both actor and partner effects were significant. For actor effect, teams’ maximum individual FWHR positively predicted the points that they negotiated, *b* = 1.14, bootstrap 95% CI = [0.33, 2.06]. For the partner effect, teams’ maximum individual FWHR negatively predicted the points their counterparts negotiated, *b* = -1.24, bootstrap 95% CI = [-2.28, -0.37]. The actor-partner interaction was not significant. These results imply that teams with higher maximum individual FWHRs perform well in value claiming rather than value creating, because value creating would have also benefitted their negotiation partners’ outcomes. The results resonate with the theory that FWHR is an indicator of dominance.

Because the actor effect was positive but the partner effect was negative, we tested whether the actor effect plus the partner effect equaled zero. To test the hypothesis, we ran another model in which actor effect plus partner effect was restricted to be zero, and then compared this model with the model without the constraint. The result showed that actor effect and partner effects had equivalent absolute values but with opposite directions, χ2(1) = 0.06, *p* = 0.799.

In sum, both the linear mixed effects modeling and APIM analyses suggest that teams of executives with larger FWHRs claim more value during negotiations.

## Discussion

Although the negotiation literature has focused mainly on dyadic negotiations, effects found at the dyadic level often cannot be observed at the intergroup level (O’Connor, 1997; Thompson et al., 1996; Morgan and Tindale, 2002). In the current study, we found that groups of Chinese executives with larger maximum individual FWHRs achieved objectively better outcomes than groups with smaller FWHRs. This positive effect of the FWHR on negotiation outcomes was largely due to the individual within the group with the largest FWHR. This evidence is the first to show that the FWHR influences negotiations between groups. While there are good reasons to suspect that the FWHR may increase or decrease negotiation performance between groups, our results suggest that having at least one negotiator with a large FWHR may enhance intergroup negotiation outcomes. This research provides one of the rare examples that stable negotiator characteristics, such as the FWHR, can impact negotiation outcomes. The present research is also the first such demonstration of the FWHR in a non-Western sample, supporting the notion that the FWHR may be a cue of universal social utility. An additional strength of this research is that we randomly assigned executives to negotiation groups, as opposed to providing correlational evidence derived from pre-existing groups.

As the FWHR is a largely static characteristic, the current findings suggest that during one-time intergroup negotiations, having (male) negotiators with relatively greater FWHRs in one’s ingroup may produce better outcomes for the ingroup, particularly if the negotiation contains a strong distributive component. In such situations, negotiators may wish to pay closer attention and possibly devote more resources to better handle specific negotiators with relatively large FWHRs on the other side of the table. Nonetheless, greater FWHRs may not be advantageous in all circumstances. For instance, in negotiations short of distributive issues, having negotiators with greater FWHRs may not help. At other times, organizational norms may value harmony and agreement over conflict and maximizing gain. In such milieus, larger FWHRs may negatively impact the negotiation process.

One limitation of the current study is that because negotiation outcomes were made at the group level, we could not assess specific individuals’ contributions to the within-group dynamics. In terms of within-group dynamics, some research shows that the FWHR predicts greater ingroup cooperation on a public goods game when outgroup competition is salient (Stirrat and Perrett, 2012). It was also not clear whether and to what extent that the negotiator with the largest FWHR played a leadership role in the ingroup. Future research could examine individuals’ contributions and the interrelationships among team members in relation to the FWHR.

The current study also does not specify the exact route of the FWHR influence on negotiation outcomes. It could be that negotiators with larger FWHRs actually behaved more aggressively and claimed more value for the ingroup. It could also be the case that negotiators of larger FWHRs did not necessarily behave in a more aggressive manner. However, the perception or lay theory that negotiators with larger FWHRs are more aggressive may have made the negotiation counterparts readily concede value. Previous research suggests that psychological mechanisms such as low perceived prosociality and high dominance prompted by the FWHR could be responsible for the established effects. Another limitation is that we were unable to examine the role of the FWHR in women. To ensure maximum inclusion of women, future research could take photographs straight on with hair away from the face. However, our sensitivity analyses revealed that including gender composition did not influence the results. Furthermore, although some studies find effects of the FWHR on dominance and aggression in women, many do not and meta-analyses show larger effects for men than women (Geniole et al., 2015). Nevertheless, future research is needed to clarify these issues with empirical evidence.

## Conclusion

In sum, the FWHR may provide useful insight into other groups’ potential negotiation behavior that is not influenced by self-presentation motivations or social desirability. Within a dynamic intergroup negotiation context, groups with men with relatively larger FWHRs achieved better negotiation outcomes than groups with men with relatively smaller FWHRs.

## Author Contributions

YY and TD designed the study. YY, CT, XQ, and CW coordinated data collection. CT, XQ, CW, and TD analyzed the data. YY and TD wrote the introduction and discussion sections. CT, XQ, CW, and TD wrote methods and results sections. XQ presented the initial findings at the Annual Meeting of the International Association for Conflict Management in 2017.

## Conflict of Interest Statement

The authors declare that the research was conducted in the absence of any commercial or financial relationships that could be construed as a potential conflict of interest.
